# Abnormal language-related oscillatory responses in primary progressive aphasia

**DOI:** 10.1016/j.nicl.2018.02.028

**Published:** 2018-03-01

**Authors:** A. Kielar, T. Deschamps, R. Jokel, J.A. Meltzer

**Affiliations:** a Rotman Research Institute, Baycrest Health Sciences Toronto, Ontario, Canada; b Department of Psychology University of Toronto, Toronto, Ontario, Canada; c Department of Speech-Language Pathology, University of Toronto, Toronto, Ontario, Canada; d Canadian Partnership for Stroke Recovery, Ottawa, Ontario, Canada

**Keywords:** MEG oscillations, Primary progressive aphasia (PPA), Sentence comprehension

## Abstract

Patients with Primary Progressive Aphasia (PPA) may react to linguistic stimuli differently than healthy controls, reflecting degeneration of language networks and engagement of compensatory mechanisms. We used magnetoencephalography (MEG) to evaluate oscillatory neural responses in sentence comprehension, in patients with PPA and age-matched controls. Participants viewed sentences containing semantically and syntactically anomalous words that evoke distinct oscillatory responses. For age-matched controls, semantic anomalies elicited left-lateralized 8–30 Hz power decreases distributed along ventral brain regions, whereas syntactic anomalies elicited bilateral power decreases in both ventral and dorsal regions. In comparison to controls, patients with PPA showed altered patterns of induced oscillations, characterized by delayed latencies and attenuated amplitude, which were correlated with linguistic impairment measured offline. The recruitment of right hemisphere temporo-parietal areas (also found in controls) was correlated with preserved semantic processing abilities, indicating that preserved neural activity in these regions was able to support successful semantic processing. In contrast, syntactic processing was more consistently impaired in PPA, regardless of neural activity patterns, suggesting that this domain of language is particularly vulnerable to the neuronal loss. In addition, we found that delayed peak latencies of oscillatory responses were associated with lower accuracy for detecting semantic anomalies, suggesting that language deficits observed in PPA may be linked to delayed or slowed information processing.

## Highlights

• Evaluated induced oscillations in patients with PPA using MEG.
• PPA patients showed delayed latencies and attenuated amplitude of responses.
• Preserved right hemisphere regions support semantic processing.
• Delayed latencies of oscillatory responses associated with impaired performance.
• Language deficits in PPA linked to delayed or slowed information processing.

## 1 Introduction

Primary progressive aphasia (PPA) is a neurodegenerative syndrome in which selective degeneration of cortical areas supporting language processing leads to a progressive impairment of language functions, with initial preservation of other cognitive domains (Mesulam, 2003; Mesulam et al., 2009). Recent diagnostic guidelines recognize three main variants of PPA (Gorno-Tempini et al., 2004, Gorno-Tempini et al., 2011; Mesulam et al., 2009, Mesulam et al., 2014): nonfluent/agrammatic, logopenic, and semantic, although considerable overlap exists between these groups, and many patients remain unclassifiable within the present guidelines (Mesulam et al., 2014; Mesulam and Weintraub, 2014; Wicklund et al., 2014). The language symptoms evinced by the three variants depend largely on the distribution and location of cortical atrophy, and there is a significant but variable link between the most strongly affected brain networks and distinct forms of molecular pathology, each variant being characterized by abnormal deposits of a different protein (Davies et al., 2005; Grossman, 2010; Gorno-Tempini et al., 2004; Mesulam et al., 2009, Mesulam et al., 2014; Snowden et al., 2007; Wilson et al., 2012). The semantic variant, with TDP-43-based neurodegeneration centered in the left temporal lobe, differs strongly from the other two, with marked impairment of single word comprehension and object naming, with relatively preserved grammatical structure and fluency (Mesulam et al., 2014). Agrammatic and logopenic PPA are more difficult to distinguish, with the former characterized by breakdown of grammatical production linked to tau protein-based degeneration of the inferior frontal gyrus, and the latter by reduced and slowed speech production, preserved grammar, and repetition deficits, linked to Alzheimer's (beta-amyloid and tau protein) pathology centered in the parietal lobe.

Although the location of neurodegeneration is thus far the best predictor of individual variation in linguistic performance across patients, alterations in network function beyond the regions of frank atrophy may provide valuable information on how the brain copes with ongoing neurodegeneration, and can help explain why some patients can maintain a high degree of function late into the disease course. Characterization of functional alterations in PPA will also inform efforts to develop effective interventions that harness the brain's capacity for adaptive plasticity, including the compensatory engagement of structurally intact networks to support language processing. Functional neuroimaging studies have the potential to reveal these characteristics of the disease and their link to cognitive preservation in PPA. Although the variants of PPA have largely been characterized by differences in language production, it is far easier to experimentally probe linguistic processing through comprehension paradigms, rather than production paradigms. Language comprehension is also strongly affected in PPA, although these deficits have received less attention than the production deficits.

In the semantic variant, comprehension can be severely compromised on the single word level, while this is usually preserved in the other variants. Most PPA patients exhibit some degree of comprehension impairment on the sentence level (Grossman et al., 1996; Grossman and Moore, 2005). A recent study investigating quantitative relationships of comprehension with patterns of cortical atrophy across PPA patients found that single-word comprehension deficits were strongly related to atrophy in the left anterior temporal lobe, characteristic of the semantic variant, whereas impaired comprehension of syntactically complex, semantically reversible sentences was associated with atrophy in both frontal and temporo-parietal regions, associated with the agrammatic and logopenic variants, respectively (Peelle et al., 2008; Mesulam et al., 2015). Interestingly, these are the same areas in which increased fMRI activation has been observed during comprehension of syntactically complex sentences relative to simpler sentences (Chen et al., 2006; Meltzer et al., 2010; Peelle et al., 2010).

Difficulties with sentence comprehension may arise at both semantic and syntactic levels, as listeners (or readers) must combine both the meaning of words and their grammatical relationships to arrive at the correct interpretation of the sentence. Results of word monitoring studies have provided evidence that sentence comprehension problems in PPA may be caused by deficits in various aspects of on-line grammatical processing (Grossman et al., 2005; Grossman et al., 2005; Peelle et al., 2007). It has been suggested that PPA patients' sentence comprehension difficulty may be due to slowed information processing speed (Grossman et al., 2005). Delayed processing of phonological information during naming has been also observed in logopenic and agrammatic PPA (Mack et al., 2015).With their high temporal resolution, electrophysiological techniques such as electroencephalography (EEG) or magnetoencephalography (MEG) can directly probe altered neural dynamics in PPA, particularly with respect to the brain's response to specific words within a sentence.

To date, only a few studies have examined electrophysiological activity related to the linguistic impairments in PPA patients, and most of them have investigated language processing at the single word level. These studies indicate that event-related potential (ERP) responses elicited by linguistic stimuli are altered in patients with PPA. For example, Giaquinto and Ranghi (2009) recorded event related potentials while participants with PPA performed a word recognition task. They found that the N400 potential, associated with word recognition, was delayed and reduced in amplitude in these patients, and progressively deteriorated until it was no longer present. Similarly, PPA patients showed abnormal N400 effects to unrelated mismatch words in an object-word matching task (Hurley et al., 2009).

### 1.1 MEG oscillatory measures of task-related activation

Pathological alterations of neural activity can be identified with high spatial and temporal resolution using MEG. This non-invasive technique detects magnetic fields at the surface of the head and can spatially localize post-synaptic currents generated in synchronously firing neuronal assemblies. Compared to EEG, MEG allows for more accurate reconstruction of source activity because magnetic fields are only minimally affected by passing through the skull, (Hamalainen, 1993). Furthermore, MEG is ideally suited for characterization and localization of oscillatory signals generated by neural activity. These signals may carry unique information useful for understanding the pathophysiology of PPA and how it affects language function.

Induced oscillations in MEG have been studied using beamforming techniques for source analysis (Vrba, 2002; Vrba and Robinson, 2001). This method estimates a virtual signal at a particular location in the brain while attenuating activity arising from other brain areas and extracranial sources, such as ocular artifacts (Cheyne et al., 2006; Robinson, 2004). Several studies with neurologically unimpaired participants identified power decreases in the alpha (8–12 Hz) and beta (15–30 Hz) ranges as a reliable indicator of increased neural activity, with close correspondence to the blood-oxygen-level-dependent (BOLD) responses in diverse parts of the cortex (Brookes et al., 2005; Hillebrand et al., 2005; Hanslmayr et al., 2012; Meltzer and Braun, 2011).

In a recent set of studies, we used MEG with beamforming to map the brain regions involved in the processing of semantic and syntactic aspects of language in healthy controls and patients with stroke-induced aphasia (Kielar et al., 2015; Kielar et al., 2016). We found that activation of specific language regions was detectable as an event-related desynchronization (ERD), or power decrease, in a broad frequency range covering both the alpha and beta bands (8–30 Hz). Processing of semantic anomalies was associated with 8–30 Hz ERD in a left-lateralized set of ventral frontotemporal regions, whereas syntactic anomalies activated both dorsal and ventral regions bilaterally. Power modulations in this frequency range have also been reported in other MEG studies examining induced oscillations to semantically or syntactically anomalous words (Bastiaansen et al., 2009; Wang et al., 2012).

Changes in language-related oscillatory responses associated with PPA have not yet been extensively investigated with MEG. In a recent study, Miller et al. (2013) studied changes in oscillatory responses during a verb generation task in one patient with semantic variant of PPA. In contrast to the left lateralized activation observed in the healthy controls, the patient with PPA showed beta band ERD localized to the right hemisphere. The possibility of compensatory linguistic processing occurring in preserved brain networks is highly relevant for intervention, as future treatments may focus on maximizing the compensatory potential of such areas through behavioral therapy, noninvasive brain stimulation, and pharmacological approaches. The engagement of right-hemisphere homologous networks in PPA is especially intriguing, given that this has been seen often in post-stroke aphasia, and has been interpreted as both adaptive (Crinion and Price, 2005; Thulborn et al., 1999) and maladaptive (Perani et al., 2003; Naeser et al., 2004) in different contexts.

### 1.2 Present study

The goal of the present study is to identify patterns of MEG induced oscillatory responses during sentence comprehension related to impairment and preservation of linguistic function in PPA. We studied both semantic and syntactic processing to identify changes in oscillatory patterns and neural recruitment associated with these different types of linguistic information. We asked whether impaired language processing in PPA is associated with altered patterns of oscillatory responses, and whether spared language functions can be associated with recruitment of preserved brain regions in the left and right hemispheres. Finally, we wanted to determine whether the magnitude and type of neural recruitment differs for semantic and syntactic processing. The design of the present study allowed us to identify relationships between neural responses to semantic and syntactic anomalies, online and offline language performance, and patterns of cortical atrophy within the PPA group. Because both agrammatic and logopenic variants are associated with sentence comprehension deficits with relatively preserved single-word comprehension, we conducted the present study in a group of patients with either diagnosis. To our knowledge, detection of semantic and syntactic anomalies has not been investigated behaviorally in PPA. Although the logopenic and nonfluent variants of PPA differ in the location of their atrophy, in temporoparietal and frontal regions respectively, atrophy in both of these regions has been linked to deficits in sentence comprehension, with the frontal regions especially implicated in comprehension of grammatically complex sentences (Peelle et al., 2008; Thompson et al., 2013). Based on this we hypothesized that anomaly detection would be impaired in both groups relative to controls, and that the variability in performance across patients would be related to differences in neural activity induced by the anomalies.

## 2 Methods

### 2.1 Participants

MEG data were acquired from 13 patients with PPA and 15 age-matched healthy controls. This study was approved by the Research Ethics Board (REB) at Baycrest Health Sciences, University of Toronto. All volunteers gave their written informed consent prior to the study and were compensated for their participation. Demographic, clinical characteristics, and cognitive scores for patients and controls are summarized in Table 1.

**Table 1:** Demographic and neuropsychological characteristics for patients and controls.

	PPA		Controls	
	Mean	*SE*	Mean	*SE*
Age(years)	69.62	*2*.*08*	69	*1*.*77*
Education(years)	14.38	*0*.*85*	17.46	*0*.*69*
Time post Onset(years)	2.54	*0*.*22*		
MOCA	19.77	*1*.*69*	27	*0*.*45*
BNT	39.84	*3*.*8*	57.07	*0*.*6*
Letter fluency	4	*0*.*5*	14	*0*.*9*
SPPT	77	*7*.*5*	99.56	*0*.*3*
SCT	84	*5*	100	*0*
PPVT	92	*2*.*6*	119	*3*.*1*
C&C	77	*2*.*9*	90	*1*.*1*
WAB flu	7	*0*.*6*		
WAB Rep	8	*0*.*5*		
WAB Comp	8	*0*.*7*		
WAB BAS	81	*3*.*6*		
WAB BLS	79	*4*.*2*		

MoCA: Montreal Cognitive Assessment (Nasreddine et al., 2005). Measured out of 30 points; Values from 30 to 26 points indicate normal performance. A score of 25 or lower (from maximum of 30) is considered significant cognitive impairment.

Explanation of Abbreviations: BNT = Boston Naming Test (score out of 60); Letter fluency: FAS (D-KEFS); SPPT = Northwestern Assessment of Verbs and Sentences Sentence Production Priming Test. NAVS_SCT = Northwestern Assessment of Verbs and Sentences-Sentence Comprehension Test, total: overall score on all sentence types; PPVT = Peabody Picture Vocabulary Test; C&C = Camel and Cactus Test (Cambridge Semantic Battery); WAB = Western Aphasia Battery: Bedside version, Flu = Spontaneous Speech Fluency, Comp = Auditory Verbal Comprehension, Rep = Repetition; BAS: Bedside Aphasia Score; BLS: Bedside Language Score.

Bedside Language Score (WAB_BLS) was determined by summing the Speech Content, Fluency, Auditory Verbal Comprehension, Sequential Commands, Repetition, Object Naming, Reading, and Writing scores, dividing the sum by 8 and multiplying the result by 10.

Bedside Aphasia Score (WAB_BAS) was determined by summing the Speech Content, Fluency, Auditory Verbal Comprehension, Sequential Commands, Repetition, and Object Naming scores, dividing the sum by 6 and then multiplying result by 10.

Italics indicate standard error of the mean *(SE)*.

Participants with PPA were recruited from several sources in Toronto, Ontario and surrounding areas. These included the Memory Clinic at Baycrest Health Sciences, the Aphasia Institute (www.aphasia.ca), and the March of Dimes Aphasia and Communication Disabilities Program (http://www.marchofdimes.ca/EN/programs/acdp/Pages/AphasiaAndCommunicationDisabilitiesProgram.aspx). Patients (7 females) ranged in age from 58 to 83 years, and had 12 to 20 years of education. All participants were right handed as measured by Edinburgh Handedness Inventory (Oldfield, 1971; Williams, 2010). They were all native speakers of English, and had normal hearing and normal or corrected to normal vision. All patients retained sufficient capacity of language comprehension to consent for the study and follow task instructions. Exclusion criteria were earlier neurological diseases, childhood language disorders, head traumas or brain surgery, epilepsy, severe psychiatric disorders, and unstable or poor health. Participants were diagnosed with PPA prior to the study by a speech language pathologist and/or board certified neurologist. PPA diagnosis was based on the basis of the convergence of the clinical presentation, narrative speech samples, and the results of standardized tests. A diagnosis of PPA required progressive deterioration of speech and/or language functions, with the deficit largely restricted to speech and/or language at the onset and throughout the early stage of the disease (first 2–3 years). All patients in this study were diagnosed as nonfluent/agrammatic or logopenic variants based on recent guidelines (Gorno-Tempini et al., 2011). Seven individuals with effortful, halting speech and/or agrammatic language were classified as the nonfluent variant, and six patients with impaired word retrieval and phrase/sentence repetition were classified as logopenic. The disease severity and general cognitive status was assessed with the Montreal Cognitive Assessment (MoCA). The mean score for patients was 19.77 points out of maximum 30 (min = 7, max = 26), indicating mild to moderately severe cognitive impairment. All participants were living at home alone or with family members.

Participants with PPA were matched with a group of healthy older controls for age, *F* < 1. All healthy volunteers were recruited from the greater Toronto area by REB approved advertisements from the University of Toronto community and from the Baycrest Health Sciences subject pool. All of the neurologically unimpaired participants (4 females) were right handed native speakers of English, and reported normal hearing and normal or corrected-to-normal vision. Participants had no history of neurological, psychiatric, speech, language, or learning disorders and none were taking neuroleptic or mood altering medications at the time of the study. Age-matched controls participated in all behavioral and neuroimaging assessments completed by the PPA patients. All older control participants tested within normal limits on all cognitive and linguistic tests.

### 2.2 Cognitive and language assessment

Prior to participation in the MEG experiment, patients and age-matched controls completed a thorough neuropsychological battery to assess several domains of cognitive and language functioning. General cognitive status was measured using the Montreal Cognitive Assessment (MoCA, Nasreddine et al., 2005). Tests of language function included the Western Aphasia Battery Revised (Bedside Record Form, Kertesz, 1982, Kertesz, 2007) for classification of aphasia type, supplemented by selected subtests of reading, spelling and repetition from the PALPA exam (Psycholinguistic Assessments of Language Processing in Aphasia, Kay et al., 1992). Verbal fluency was assessed using Letter fluency tests (Letters FAS) from the Delis-Kaplan Executive Function System (D-KEFS, Delis et al., 2001), and confrontation naming was examined using the 60-item Boston Naming Test (BNT, Kaplan et al., 2001). Comprehension and production of sentences varying in syntactic complexity were assessed using two subtests of the Northwestern Assessment of Verbs and Sentences (NAVS, Thompson, 2011; Cho-Reyes and Thompson, 2012), respectively, the Sentence Comprehension Test (SCT) and the Sentence Production Priming Test (SPPT). Receptive lexical semantics and vocabulary knowledge were examined using the Peabody Picture Vocabulary Test, Fourth Edition (Dunn and Dunn, 2007). Semantic knowledge independent of expressive verbal abilities was assessed using the Camel and Cactus Test (Adlam et al., 2010).

For further characterization of cognitive status, and to rule out the possibility that nonlinguistic deficits could account for low performance on an MEG comprehension task, participants were administered tests of episodic memory, executive function, and visuospatial abilities. Episodic memory was assessed using word lists (immediate and delayed free and cued recall, and recognition) from the Kaplan Baycrest Neurocognitive Assessment (KBNA, Leach et al., 2000) and the logical memory subtest from WMS-IV (immediate, delayed recall, and recognition, Wechsler, 2009). Non-verbal episodic memory was tested using the Facial Recognition Test (Warrington, 1984). In addition, executive functions were examined using the forward and backward digit span tests from the WAIS-IV, and the Trail Making Test (Trails A and B) from D-KEFS.

Visuospatial abilities were assessed using the Complex Figure subtest from KBNA (immediate: copy and recall, delayed: recall and recognition), Symbol Cancelation also from KBNA, and a short form of Benton's Judgement of Line Orientation Test (Calami et al., 2011; Benton et al., 1983). Mean scores and standard errors for patients and controls on selected measures are presented in Table 1.

### 2.3 Using PCA to derive a language measure

In this study, we sought to characterize associations between altered neural responses to linguistic stimuli and linguistic impairment, both in terms of the performance on the sentence comprehension task performed during MEG, and broader measures of linguistic ability based on the cognitive battery. To summarize the overall degree of language impairment, we entered the scores from individual tests, from patients and controls combined, into a Principal Component Analysis (PCA) with Varimax Rotation. Given that the cognitive battery was heavily weighted towards tests of language, it was expected that the first component returned from PCA would serve as an overall measure of language impairment, separated from other cognitive domains, thus serving as a convenient means of dimensionality reduction. The MoCA and WAB were excluded from the PCA, as they are multifactorial tests covering a wide range of abilities.

PCA and related analyses were carried out with SPSS version 22 (IBM). The test scores were normalized by converting them to z-scores, controlling for the scale differences between the various tests. In order to increase the proportion of cases to variables for the PCA analysis, the control group included 15 age-matched controls who took part in the MEG study, plus an additional 16 age-matched older controls who completed the same neuropsychological battery. A scree-plot analysis of the variance accounted for by each component suggested that the test scores clustered into two principal components, and the loadings indicated that one component reflected language measures and the second reflected memory and visuo-spatial abilities. The test scores entered into the PCA and factor loadings are presented in Table 2.

**Table 2:** Results of Principal Component Analysis for the Neurocognitive Test Battery.

Cognitive Tests	Component loadings	
	Component 1 (*Language*)	Component 2 (*Memory* & *visuo*-*spatial*)
NAVS_vnt	0.819	
sentence_rep_b	0.813	
lm1_recall	0.802	
NAVS_sct	0.792	
NAVS_sppt	0.791	
animals_fluency	0.764	
sentence_rep_a	0.757	
BNT	0.710	
digit_backward	0.705	
digit_forward	0.692	
letter_fluency	0.664	
PPVT	0.640	
trails_B	0.631	
CNRT	0.578	
lm2_recall	0.556	
lm2_recognition	0.531	
NAVS_aspt	0.497	
cf2_recall		0.786
cf1_recall		0.784
cf1_copy		0.784
symbol cancelation		0.754
trails_A		0.727
wl2_recall		0.701
camel & cactus		0.680
NAVS_vct		0.676
cf2_recognition		0.667
wl1_recall		0.578
wl2_recognition		0.575
Facial recognition		0.404
jlo		0.370

Varimax rotation was applied to the components. Factor loadings below 0.35 are suppressed from the display. Component 1 (language) eigenvalue = 12.77, percent variance explained = 42.58; Component 2 (Memory & visuo-spatial) eigenvalue = 3.39, percent variance explained = 11.29.

Explanation of Abbreviations: animals_fluency = Category Fluency (D-KEFS); BNT = Boston Naming Test (score out of 60); Camel & Cactus = Camel and Cactus Test (Cambridge Semantic Battery); CNRT: Children's Nonword Repetition Test; cf. = Complex Figure Drawing (KBNA); digit forward and backward = Digit Span (WAIS-IV); JLO = Benton's Judgement of Line Orientations; Facial recognition = Facial Recognition Test; letter_fluency = Letter Fluency (D-KEFS); lm = Logical Memory (WMS-IV); NAVS = Northwestern Assessment of Verbs and Sentences (vnt: verb naming test, sct: sentence comprehension test, aspt: argument structure production test, sppt: sentence production priming test, vct: verb comprehension test); PPVT = Peabody Picture Vocabulary Test; symbol cancelation = Symbol Cancelation (KBNA); trials = Trail Making Test (D-KEFS, Trails A and B); sentence_rep = Sentence Repetition (Aphasiabank); wl = Word Lists (KBNA).

Subsequently, factor scores on each component were calculated for each participant and used to test for correlations with MEG activation (reflected by 8–30 Hz ERD) for semantic and syntactic anomalies. To test whether scores derived from the two principal components differentiated between patients and controls, we performed repeated measures ANOVA with factor type (language component, memory component) entered as a repeated-measures variable and group (PPA, age-matched) as a between-subjects variable. The analysis revealed a significant main effect of group, *F*(1,42) = 44.44, *p* < .001, and a significant factor type by group interaction, *F*(1,42) = 6.19, *p* = .017. Post-hoc tests of this interaction indicated that the language component differentiated patients from controls, *t*(42) = 5.56, *p* < .001, whereas there was no significant difference between groups on the memory and visuo-spatial component, *t*(42) = 1.25, *p* = .232.

### 2.4 Sentence comprehension task

All participants completed a visual sentence-judgement task during MEG acquisition. More detailed description of the sentence materials and procedure can be found in our previous papers reporting results from young healthy controls and patients with post-stroke aphasia (Kielar et al., 2015, Kielar et al., 2016). A brief description of the paradigm is given below. Examples of experimental sentences are presented in Table 3.

**Table 3:** Example sentences used in the experiment. The critical words in correct and anomalous sentences are underlined.

Code	Condition	Example sentences
COR	Correct	She will go to the bakery for a loaf of bread
SEM	Semantic anomaly	She will go to the bakery for a loaf of books
SYN	Syntactic anomaly	She will going to the bakery for a loaf of bread

Each trial started with a 500 ms fixation cross, followed by word-by-word presentation of the sentence. The words were presented in white font on a black background in the center of the screen. Each word appeared for 350 ms, followed by a blank screen for 400 ms. The last word of the sentence was followed by a blank screen of 2500 ms, after which a response prompt (a question mark) was presented. At this point participants performed a button-press judgement on whether the sentence was correct (i.e., free of semantic and syntactic errors), or “unacceptable”. All participants indicated correct sentences with their right index finger, and incorrect sentences with their right middle finger. Participants were instructed to withhold their button-press judgement until the response cue appeared. Visual stimuli were displayed on a screen approximately 0.5 m from the participant's face, projected via mirrors from an LCD projector placed outside the magnetically shielded room to avoid interference. In order to become familiar with the procedure, participants first completed a practice block of six sentences.

### 2.5 MRI scans acquisition and processing

MRI data were always acquired after the MEG session, either the same day or up to two weeks after. MRI scans were acquired on a 3-Tesla scanner (Siemens TIM Trio) with 1 mm isotropic voxels, TR = 2000 ms, TE = 2.63 ms, FOV = 256 × 256 mm, 160 axial slices, scan time 6 min, 26 s. The MPRAGE image was used to construct a head model for MEG source modeling and Voxel Based Morphometry (VBM). MR-visible markers were placed at the fiducial points for accurate registration, aided by digital photographs from the MEG session. T1 images were skull stripped in AFNI software (Cox, 1996).

### 2.6 Voxel-based morphometry: Image processing and analysis

Voxel-based morphometry (VBM) implemented in SPM8 software (Wellcome Department of Cognitive Neurology, London, UK), was used to derive segmented, smoothed and normalized gray matter maps for PPA patients and age-matched controls. Before processing, T1 images were evaluated for quality and manually repositioned so that the anterior commissure was set as the origin. This was done to ensure consistent starting estimates for the unified segmentation routine. One patient's scan was of insufficient quality due to excessive motion artifacts and was excluded from the analysis.

T1-weighted structural images were spatially normalized, bias corrected, and segmented into gray matter, white matter, and cerebrospinal fluid using the unified segmentation algorithm implemented in the “new segment” option of SPM8 (Ashburner and Friston, 2005). The accuracy of inter-subject alignment was increased by performing nonlinear image registration procedures implemented in the DARTEL (Diffeomorphic Anatomical Registration through Exponential Lie algebra) toolbox (Ashburner, 2007; Ashburner and Friston, 2009). For each participant, flow fields were calculated during template creation that contained the nonlinear deformation information on the native image transformation to the template. These flow fields were applied to each participant's image. Next, the final template was registered to MNI space using an affine transform and this transformation was incorporated into the warping process, so that the individual spatially normalized scans could be brought into the common MNI space. During this final normalization step, the gray and white matter probability maps were scaled by their Jacobian determinants and smoothed using a 10 mm FWHM isotopic Gaussian kernel. An estimate of total intracranial volume (TIV) was computed by combining the gray matter, white matter, and cerebrospinal fluid segments derived from the segmentation step.

To identify cortical atrophy at the group level, the images for the PPA patients were compared with 25 age matched controls using an independent sample *t*-test, with age, sex, and total intracranial volume included as covariates. The control group for VBM analysis consisted of the 15 age-matched controls who took part in the MEG study, plus an additional 10 age-matched older controls.

### 2.7 MEG acquisition and analysis

MEG signals were recorded with a 151-channel whole-head system with axial gradiometers (VSMMedTech, Coquitlam, Canada). MEG was recorded continuously at a sampling rate of 625 Hz, and acquired with online synthetic 3rd-order gradient noise reduction (Vrba and Robinson, 2001). Continuous signals were cut into epochs surrounding the critical word presentation times. Head position with respect to the MEG helmet was monitored using three coils placed at anatomical landmarks of the head (nasion, left and right pre-auricular points). The head position was measured before and after each run, and averaged across runs for source analysis.

To construct head models for MEG analysis, the MR-visible markers were manually identified on the T1 image and used to mark locations of the fiducial points in AFNI. The T1-weighted MRI was spatially transformed into the coordinate space of the MEG data. The skull was stripped using Brain Extraction Tool, and a 3-D convex hull approximating the inner surface of the skull was constructed using the software package Brainhull (http://kurage.nimh.nih.gov/meglab/Meg/Brainhull). Taking into account the position of the head relative to the sensors, a multi-sphere model (Huang et al., 1999) was computed for each MEG session. To normalize MEG source estimates into MNI space, we computed a nonlinear warp of each subject's brain to a single-subject template, the “colin27” brain, using the software package ANTS (Avants et al., 2011). This warp was then used to transform single-subject MEG activity maps into MNI space.

Raw MEG sensor signals were screened for artifacts, and trials containing obvious signal disruptions were rejected (e.g., coughs, sneezes, yawns, head movements; <1% of all trials). Further signal analysis was conducted in source space using SAM beamforming (Cheyne et al., 2007; Vrba, 2002; Vrba and Robinson, 2001).

### 2.8 Task-related MEG analysis

#### 2.8.1 Time-frequency analysis on virtual channels

For initial characterization of the time-frequency dynamics induced by the paradigm in patients with PPA and age-matched control group, we analyzed activity in 90 virtual channels placed in a priori locations throughout the brain. Using the macroanatomical brain parcellation of Tzourio-Mazoyer et al. (2002), consisting of 90 cortical and subcortical regions (e.g., left superior temporal gyrus, left putamen, etc.), we took the center of each region and warped it into the coordinate space of each subject's MEG data.

We conducted time-frequency analysis on these virtual channels in source space, computing virtual signals as a product of beamforming weights and the sensor data. The beamforming weights for virtual channels were computed with Synthetic Aperture Magnetometry (SAM), using the MRI-derived head model and the data covariance matrix in a broad time-frequency window (bandwidth 0–100 Hz, time −1 to +4 s) for the critical verb for syntactic anomalies and their correct condition, and the final word for semantic anomalies and the corresponding correct condition. This allowed us to identify time periods and frequency ranges that were maximally responsive to the contrasts of interest.

We performed the time-frequency analysis on the virtual channel signals using EEGLAB software (version 9.0.4.5) running in the Matlab 2010 (v 7.6) environment. Single-trial epochs were analyzed using a moving window short-time Fourier transform with 200 overlapping time windows per trial. The length of the time window in the spectrogram analysis was 0.512 s (320 samples at a sampling rate of 625 Hz). Values at each time-frequency point were averaged over trials of each specific condition. The average log-power in the baseline period for all three conditions was used as a common baseline, subtracted from log-power at each time-frequency point, yielding the measure conventionally known as “event-related spectral perturbation,” or ERSP (Makeig, 1993). This procedure ensured that the same baseline power values were used across all conditions; thus, any differences between conditions could not be attributable to differences in the baseline. Analysis of source space virtual channels is an alternative to analyzing the raw sensor data, with the additional advantage of artifact reduction. The initial stage of virtual channel analysis served to identify the time and frequency windows in which induced oscillations occurred for patients and control groups (see results). Also, the latency of responses at specific channels (see results) was measured using the publicly available PeakFinder Toolbox in Matlab (https://terpconnect.umd.edu/~toh/spectrum/PeakFindingandMeasurement.htm).

#### 2.8.2 Whole-brain mapping of oscillatory responses

Guided by the results of the time-frequency analysis on the virtual channels and our previous findings with controls and stroke patients using the same paradigm (Kielar et al., 2015, Kielar et al., 2016), we generated whole-brain maps of oscillatory responses using SAM beamforming. These maps were computed in a specific frequency range (8–30 Hz, comprising the alpha and beta bands) and time window (0.4–1 s relative to critical word onset) to test for the statistical significance of power changes throughout the brain. For each subject, at a regular grid of locations spaced 7 mm apart throughout the brain, we computed the pseudo-T value, which is a normalized measure of the difference in signal power between two time windows (Vrba and Robinson, 2001). Correct trials were compared with each anomaly condition within the same time window relative to word onset. Due to this “dual-state” analysis approach, multi-subject statistical maps were derived from subtractive contrast images computed on the single-subject level, not from individual conditions. Beamformer weights for this analysis were computed from data within specific time and frequency windows, providing greater spatial resolution than the nonspecific weights derived from broadband data that we used for the virtual channel time-frequency analysis (Brookes et al., 2008).

Maps of pseudo-T values throughout the brain were spatially normalized to MNI space by applying the nonlinear transforms computed by ANTS (by warping the T1-weighted MRI to an MNI template), enabling random-effects analysis at the group level.

Group statistics on SAM results were computed using parametric statistics. For each experimental comparison, the spatially normalized whole-brain map of pseudo-T values was submitted to a voxel-wise one-sample *t*-test across subjects. All statistical tests were two-tailed. To correct for multiple comparisons across the whole brain, resulting statistical maps were subjected to voxel-wise thresholding and a minimum cluster-size criterion of 86 voxels, resulting in a cluster-wise corrected family-wise error rate of *p* < .05. The cluster size criterion was determined by Monte Carlo simulations conducted in the AFNI program 3dClustSim, with a voxel-wise threshold of *p* < .01. For comparisons with stronger effects (e.g., syntactic violation – correct), we used stricter voxel-wise threshold of *p* = .001. The simulations in 3dClustSim also require an estimate of the smoothness (FWHM: full width at half maximum) of the data in the absence of a true effect. For this, we computed “null” SAM maps by comparing the prestimulus intervals for two different conditions, which should not differ. Two null maps were computed for each subject for each frequency band. Smoothness estimates of these maps ranged from 18.74 to 21.77 mm, and the mean value of 20.25 mm was used in the simulations.

## 3 Results

### 3.1 Behavioral results: Sentence comprehension performance

The reaction time for two participants could not be recorded due to a response button malfunction. In this MEG study, participants were required to press button A when the sentence was correct and button B when the sentence contained an anomaly. Because the overall error rate on the sentence comprehension task could be influenced by participants' response bias, we used a signal detection method to estimate individual participants' ability to discriminate targets (anomalous sentences) from non-targets (correct sentences). Using each participant's proportion of hits (pressing button B after the presentation of an anomalous sentence) and proportion of false alarms (pressing button B after the presentation of a correct sentence) we calculated d’ values separately for semantic and syntactic anomalies. This procedure provides an estimate of performance on each sentence type corrected for response bias. Higher d’ values reflect a greater discrimination sensitivity to anomalies and, thus, a better ability to discriminate semantic and syntactic anomalies from correct sentences. We used d’-prime values for behavioral analyses, and to examine correlations between task performance and 8–30 Hz ERD.

**Fig. 1:**
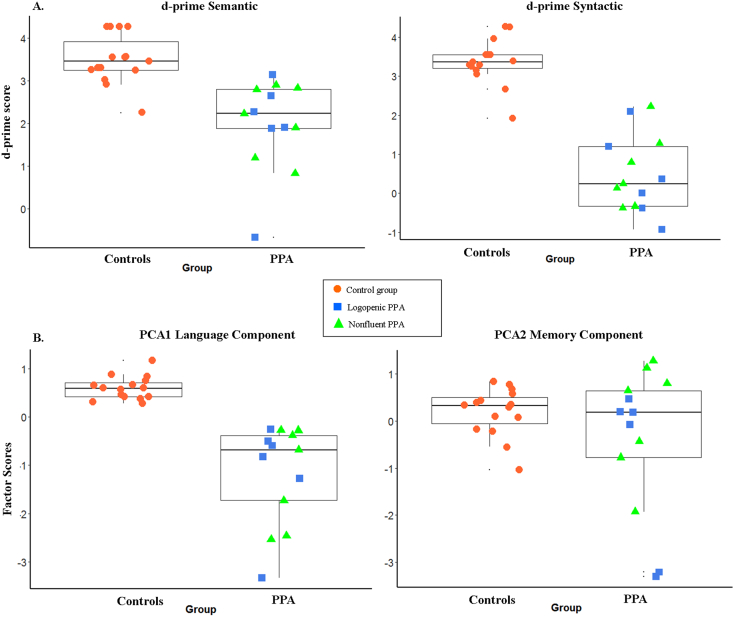
Individual scores for patients (PPA) and age matched controls (AM). Individual control participants, logopenic PPA, and nonfluent PPA are indicated with different symbols. (A) D-prime values for semantic anomalies. (B) D-prime values for syntactic anomalies. (C) Factor scores for the language component. (D) Factor scores for the memory and visuo-spatial component. The box plots show the group median, interquartile range, and full range as well as each individual score.

Behavioral results for the control group (*n* = 15) and PPA patients (*n* = 13) are presented in Table 4, including the raw percent correct and the bias-adjusted d’ values. The PPA group in this study was composed of both nonfluent and logopenic subtypes. To investigate whether any differences in performance were present for detecting semantic and syntactic anomalies, we compared behavioral scores and reaction times for the two PPA subtypes using one-way ANOVA. The analysis indicated that there were no significant differences in accuracy (d-prime semantic, d-prime syntactic, *F*s < 1) or reaction time (*Fs* < 1) between nonfluent and logopenic PPA patients. The d’ values for individual patients and controls are plotted in Fig. 1 A, which illustrates that nonfluent and logopenic patients showed similar task accuracy. As shown in Fig. 1 B, the two PPA subtypes also did not differ on the factor scores for language (*F* < 1) and memory (*F*(1, 12) = 1.636, *p* = 0.227) components obtained from the PCA analysis. Because the two PPA subtypes showed similar average performance in the critical online and offline measures of language ability, with considerable individual variability within each group, we combined them together in the subsequent analyses of MRI and MEG data.

**Table 4:** Mean percent accuracy (standard error of the mean) and reaction time in milliseconds (standard error of the mean) on the sentence comprehension task for age-matched control group (*n* = 15), and PPA patients (*n* = 13, logopenic and nonfluent variants combined).

Group	Condition			
		Accuracy%(SE)	RT(SE)	d’
Control	COR	93.24(1.24)	688.61(51.56)	
	SEM	96.8(0.70)	643.27(51.80)	3.50(0.15)
	SYN	94.27(1.43)	614.58(50.58)	3.37(0.15)
PPA	COR	77.35(3.23)	979.86(80.62)	
	SEM	81.75(6.24)	933.22(67.01)	1.98(0.29)
	SYN	39.12(7.74)	1017.17(78.22)	0.48(0.27)

d’ statistic: accuracy corrected for response bias calculated separately for semantic and syntactic anomalies. Higher value indicates better sensitivity to discriminate violations from correct sentences.

COR: correct sentences; SEM: semantic anomalies; SYN: Syntactic anomalies.

To examine behavioral differences between PPA patients and controls, accuracy (d’ values) and reaction time (RT) data were entered into separate repeated-measures analyses of variance (ANOVAs). The anomaly condition (semantic or syntactic) was entered as a within-subjects variable and the participant group – PPA patient (PPA) vs. controls – was entered as a between-subjects variable.

The analysis of accuracy data revealed a significant main effect of anomaly condition, *F*(1, 26) = 26.72, *p* < .001, a significant main effect of group, *F*(1, 26) = 69.47, *p* < .001, and a significant condition by group interaction, *F*(1, 26) = 19.08, *p* < .001. To investigate the source of this interaction, a separate post-hoc ANOVA with anomaly condition as a within-subjects variable was performed for each participant group. For PPA participants there was a significant main effect of condition, *F*(1, 12) = 21.12, *p* = .001, indicating larger d’ prime (higher accuracy) for detecting semantic anomalies than for syntactic anomalies. However, for age-matched controls there was no significant main effect of anomaly condition, *F*(1, 14) = 2.57, *p* = .131, indicating similar performance for detecting both kinds of anomalies.

We also assessed the reaction time (RT) pattern for the two participant groups and anomaly conditions (COR, SEM, SYN). For the RT data, the ANOVA with anomaly condition as a within-subjects variable and group as a between-subjects variable revealed no significant main effect of anomaly condition, *F*(2, 48) = 1.32, *p* = .276, and no significant group by condition interaction, *F*(2, 48) = 2.58, *p* = .086. However, there was a significant main effect of group, *F*(1, 24) = 16.13, *p* = .001, indicting longer reaction times for patients than controls. Note that because the behavioral response was elicited by a visual cue several seconds after the end of the sentence, RT can not necessarily be interpreted as reflecting the speed of the brain's response to the linguistic anomaly. However, latency of the MEG responses can be interpreted as such.

### 3.2 MEG results

#### 3.2.1 Time-frequency results on the virtual channels

**Fig. 2:**
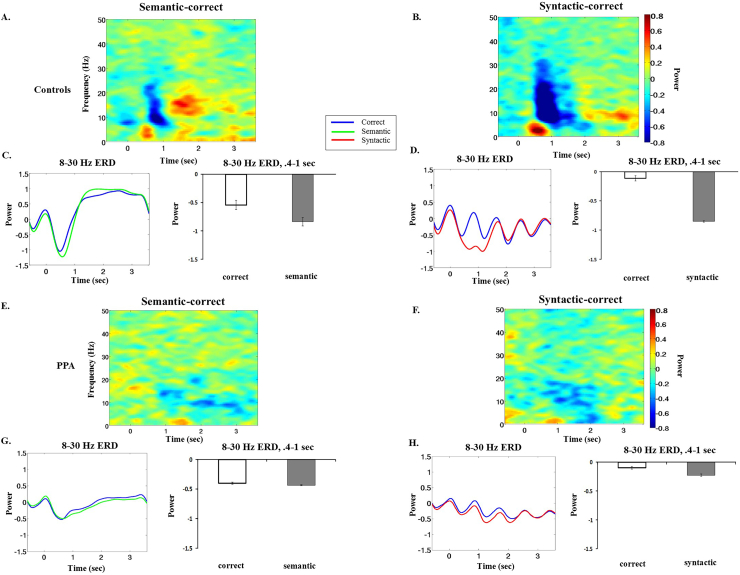
Time-frequency dynamics of SAM virtual signals averaged across 38 left hemisphere cortical channels. Age matched controls (AM, *n* = 15). (A) Time-frequency subtraction of semantic violation – correct words. (B) Time-frequency subtraction of syntactic violation – correct words. (C) Average time course of power in the 8–30 Hz band, for semantic violation and correct conditions. The bar plots show mean 8–30 Hz Event-related Desynchronization (ERD) and standard errors for each condition, in the main analysis time window from 400 to 1000 ms. (D) Average time course of power in the 8–30 Hz band, for syntactic violation and correct conditions. PPA patients (PPA, *n* = 13). (E) Time-frequency subtraction of semantic violation – correct words. (F) Time-frequency subtraction of syntactic violation – correct words. (G) Average time course of power in the 8–30 Hz band, for semantic violation and correct conditions. (H) Average time course of power in the 8–30 Hz band, for syntactic violation and correct conditions.

To get a general overview of the timing and frequency of oscillatory responses present in the data, we averaged the results of the time-frequency analysis across all 38 left cortical virtual channels. For healthy age-matched controls we observed strong oscillatory responses for both kinds of violations compared to their corresponding correct words (Fig. 2A–B), reflected in power decrease (ERD) in the 8–30 Hz range, with an onset around 0.4 s and extending in time past 1 s. The time and frequency distribution of these responses are very similar to what we observed in young controls using the same paradigm (Kielar et al., 2015). The time-course of these effects can be observed by averaging across frequencies within the specified bands and plotting the two conditions as lines (Fig. 2C–D).

For patients with PPA, we also observed power decreases in response to semantic and syntactic anomalies. Compared to controls, responses for patients were attenuated and had a later onset (Fig. 2E–F). For semantic anomalies, ERD responses were observed from about 8–20 Hz with onset around 0.9 s and lasting till about 3 s post-stimulus onset. For syntactic anomalies, ERD responses were found in the 8–20 Hz frequency range, with onset at 0.5 s and lasting till about 2 s post-stimulus onset. The time-course of these effects for PPA patients can be observed in Fig. 2G and H.

Based on the observed responses in virtual channels for the healthy age-matched controls and our previous results with young participants (Kielar et al., 2015), we used a time window of 0.4–1 s and 8–30 Hz frequency range for both semantic and syntactic anomalies, as the main analysis window for whole-brain comparisons of oscillatory responses. Healthy controls showed fairly continuous ERD across this entire range, and this time-frequency window encompassed most of the responses for semantic and syntactic anomalies.

#### 3.2.2 SAM localization of oscillatory responses

Using the selected time-frequency windows, we applied whole-brain SAM in order to localize brain responses to semantic and syntactic anomalies in PPA participants and the age-matched control group. Informed by our previous results with the same paradigm, neural “activation” is indicated by power decrease, or event-related desynchronization (ERD), in the 8–30 Hz range. Power decreases are mapped in a blue color scale on the surface of a standard reference brain in MNI space, while power increases (associated with reduced neural activity) are mapped in a yellow-red color scale. To correct for multiple comparisons at a cluster-wise level of *p* < .05, the statistical maps were thresholded at a voxelwise value of *p* = .01 or less and subjected to a minimum cluster size of 86 voxels (as described in methods). The 8–30 Hz ERD maps for nonfluent and logopenic subtypes are presented separately in Supplementary Fig. S1. The supplementary figures are shown at an uncorrected threshold to illustrate regions of overlap for nonfluent and logopenic subgroups.

**Fig. 3:**
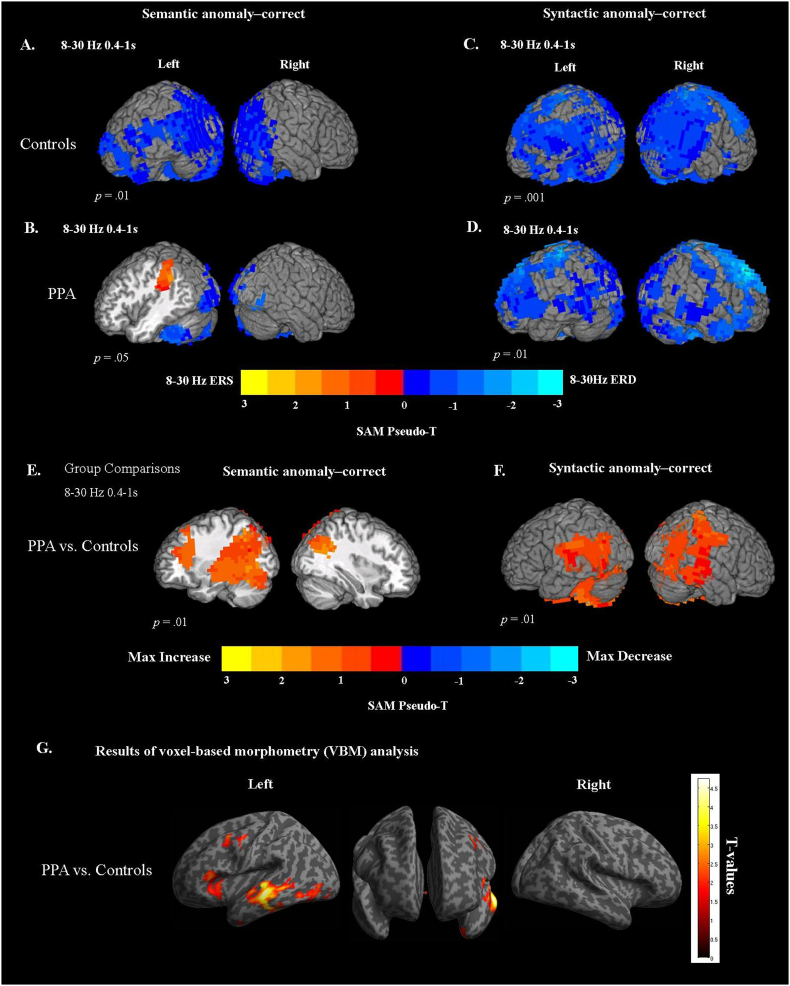
Synthetic aperture magnetometry (SAM) maps of power changes in the 8–30 Hz frequency range and 0.4–1 s time window after critical word onset for healthy age-matched controls (AM) and participants with PPA. Statistical maps were thresholded at a minimum cluster-size criterion of 80 voxels and *p* < .01. (A) Power changes for semantic anomalies vs. correct words for age-matched controls. (B) Power changes for semantic anomalies vs. correct words for PPA patients. (C) Power changes for syntactic anomalies vs. correct words for age-matched controls. (D) Power changes for syntactic anomalies vs. correct words for PPA patients. Between-group voxel-wise contrast maps of power changes in the 8–30 Hz frequency range and 0.4–1 s time window after critical word onset. Statistical maps were thresholded at a minimum cluster-size criterion of 80 voxels and *p* < .01. (E) Subtraction map for PPA patients minus age-matched controls (AM) on semantic anomalies (semantic anomalies – correct sentences). (F) Subtraction map for PPA patients minus age-matched controls on syntactic anomalies (syntactic anomalies – correct sentences). (G) Results of Voxel-based morphometry (VBM) analysis comparing gray matter volume in PPA patients (*n* = 13) versus controls (*n* = 25). The map shows regions of significant gray matter loss for PPA patients. To illustrate the regions of gray matter damage, the statistical maps were set at the lowered voxelwise threshold of *p* < .05, and corrected for multiple comparisons by controlling the family wise error (FWE) at the cluster level *p* < .05.

**Semantic Responses: 8**–**30 Hz ERD.** Fig. 3 A shows maps of 8–30 Hz ERD for semantic anomalies minus correct words in older, age-matched control participants in the main analysis window (8–30 Hz and 0.4–1 s). The comparison of semantic anomalies vs. correct words produced 8–30 Hz ERD in the occipital cortex bilaterally, including middle occipital gyrus (BA 18), primary visual cortex (BA 17), cuneus (BA 18/19), lingual gyrus (BA 19), inferior occipital and fusiform gyri (BA 37). From occipital areas, power decreases extended bilaterally to the posterior superior temporal (BA 22) and inferior and superior parietal lobules, including angular gyrus (BA 39), supramarginal gyrus (BA 40), and precuenus (BA 7). In the left hemisphere, power decreases were observed along the temporal cortex including inferior (BA 20), middle (BA 21), and superior temporal (BA 22) gyri. In the frontal regions, 8-30 Hz ERD was found in the left middle and dorsolateral frontal gyri (BA 46, 10), and extended into the most of the inferior frontal gyrus (BAs 45/44/47).

Fig. 3B presents the average ERD maps for semantic anomalies minus correct words for PPA participants in the main analysis window (8–30 Hz and 0.4–1 s). Unexpectedly, the comparison of semantic anomalies vs. correct words produced 8–30 Hz event-related synchronization (ERS), or *power increase*, in the left posterior parietal regions, including supramarginal and angular gyri (SMG, BA 40/39), and left superior parietal lobule (BA 7). In the same regions, controls showed power decreases. Subsequent analysis of ERD peak latency in this region (see below) suggested that this difference is mainly attributable to a longer onset latency of the ERD response in PPA patients, rather than a true reversal of sensitivity from ERD to ERS. As with age-matched controls, 8–30 Hz power decreases were observed in the bilateral occipital gyri (BA 17/18), including cuneus (BA 18/19), lingual gyrus (BA 19), fusiform gyrus (BA 37), and cerebellum.

**Syntactic Responses: 8**–**30 Hz ERD.** For older controls, comparison of syntactic anomalies with correct words in the main analysis window (8–30 Hz and 0.4–1 s) produced widespread power decreases in both left and right hemispheres (Fig. 3C). The 8–30 Hz ERD involved bilateral middle and inferior occipital gyri (BA 19/18), lingual gyrus (BA 18), fusiform gyrus (BA 37), and cuneus. 8–30 ERD was also found in the left and right superior occipital gyri (BA 19) and precuneus (BA 7). The responses extended bilaterally into the superior and inferior parietal lobule, including angular (BA 39) and supramarginal gyri (BA 40). Along the temporal cortex 8–30 Hz ERD power decreases were observed in the inferior (BA 20), middle (BA 21), and superior temporal (BA 22) gyri. Power decreases were also observed along the precentral (BA 6/8) and postcenral gyri (BA 3, 2, 5), including motor cortex (BA 4), premotor, and supplementary motor areas (BA 6). The 8–30 Hz ERD extended to most of the inferior frontal gyrus (BA 44/45 and 47), and middle and superior frontal cortex (BA 9 and 8), bilaterally.

Fig. 3D presents the average ERD maps for syntactic anomalies minus correct words in PPA participants in the main analysis window (8–30 Hz and 0.4–1 s). Similar to age-matched controls, patients showed extensive bilateral 8–30 Hz power decreases in response to syntactic anomalies, including middle occipital gyri (BA 19), cuneus (BA 17/18), lingual gyrus (BA 19), and fusiform gyri (BA 37). In the temporal cortex significant 8-30 Hz ERD responses were found bilaterally in the left superior (BA 22) and in the middle (BA 21) temporal gyri, extending into the superior and inferior parietal lobules (most of supramarginal gyrus (BA 40), angual gyrus (BA 39), and precuneus, BA 7). Power decreases were also observed along the middle and superior frontal gyri (BAs 8/9), dorsolateral frontal cortex (BAs 49/10), and extended into the precentral (BA 6/8) and postcentral gyri (BAs 3, 2), motor cortex (BA 4), premotor, and supplementary motor areas (BA 6), including most of the inferior frontal gyrus (BA 44/45/47) in both left and right hemispheres.

#### 3.2.3 Group comparisons of ERD responses

**Comparison of Semantic Responses: 8**–**30 Hz ERD.** Fig. 3E displays the subtraction map for patients minus age-matched controls for responses to semantic anomalies (semantic anomalies minus correct sentences) in the 8–30 Hz frequency range and 0.4–1 s time window. As ERD is a negative quantity, the warm colors in Fig. 3E indicate attenuated ERD in patients compared to controls. These maps show reduced ERD for patients in the left parietal areas, including inferior and superior parietal lobules and precuneus (BAs 40, 39, 7), The group differences extended into the left middle and superior temporal areas (BA 21 and 22), left fusiform (BA 37), and superior occipital gyri (BA 19). In comparison to older controls, patients with PPA showed diminished 8–30 Hz ERD in the left middle and superior frontal gyri (BAs 46, 9, 10). Reduced ERD was also found in the right superior parietal lobule (BA 7).

**Comparison of Syntactic Responses**: **8**–**30 Hz ERD.** The group comparison maps of brain responses to syntactic anomalies (syntactic anomalies minus correct sentences) for patients vs. age-matched controls are presented in Fig. 3F. These maps show reduced ERD for patients in the bilateral posterior middle and superior temporal gyri (BA 21, 22), along the inferior temporal gyrus (BA 20), and in the fusiform gyrus (BA 37). The group differences were also found in the inferior and superior parietal lobules, including angular (BA 39) and supramarginal gyri (BA 40), bilaterally.

### 3.3 Gray matter volumes in PPA patients in comparison to healthy controls

The results of the independent sample *t*-test comparing gray matter volumes in PPA patients versus the age-matched control group are shown in Fig. 3G. Because the sample size was small, to illustrate regions of atrophy in patients the statistical maps were thresholded at the lowered voxelwise threshold of *p* < .05, and corrected for multiple comparisons by controlling the family wise error (FWE) at the cluster level *p* < .05 (Woo et al., 2014). Thus, the analysis is only sensitive to larger clusters of atrophy. The results of VBM analysis for each subgroup are presented separately in the Supplementary Fig. S1E and F.

Patients showed areas of reduced gray matter within the left hemisphere perisylvian language network, affecting inferior frontal gyrus (BA 45/44) and middle frontal areas (BA 46, 8 and 6). The areas of reduced gray matter volume extended into the left middle and superior temporal gyri (BA 21 and 22) and posterior inferior temporal gyrus (BA 20), including fusiform gyrus (BA 37). These regions included left IFG (BA 44/45) for semantic anomalies, and portions of the left middle temporal gyrus (BA 21/22) for both semantic and syntactic anomalies. A conjunction analysis revealed that there was no overlap in voxels showing significant cortical atrophy in the PPA group, and voxels showing significant 8–30 Hz ERD responses in this group.

### 3.4 MEG 8–30 Hz ERD correlations with sentence comprehension task performance

PPA patients showed great difficulty with identifying syntactic anomalies, while they performed relatively well on semantic anomalies and correct sentences (although still worse than controls). As shown in Fig. 1A and B, patients exhibited substantial variability in their performance. To examine the relationship between comprehension performance and 8–30 Hz event-related desynchronization (ERD) for the two anomaly types, we performed voxel-wise rank-order correlations (Spearman's Rho) across patients between the accuracy scores (quantified using d’) and ERD in the 8–30 Hz range. Note that because ERD is a negative quantity, most of the observed correlations are negative, reflecting that greater 8–30 Hz ERD corresponds to better performance.

**Fig. 4:**
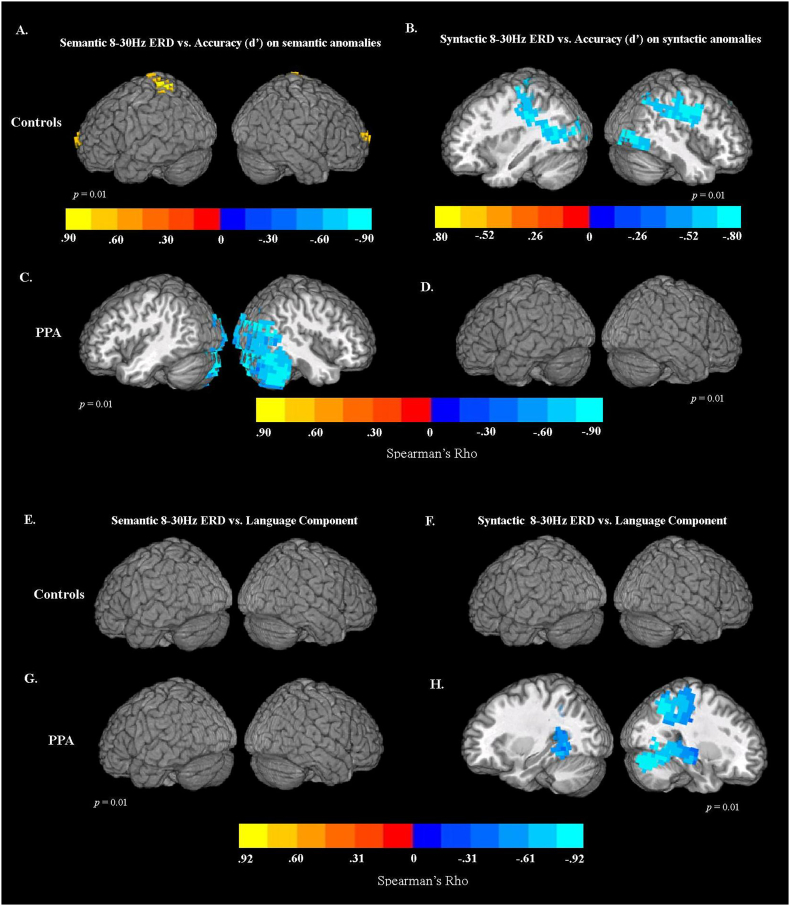
Correlations (Spearman's Rho) between MEG task activity and accuracy scores (quantified with d’) for controls and patients. Statistical maps were thresholded at a minimum cluster-size criterion of 80 voxels and *p* < .01. (A) Correlations between 8-30 Hz ERD for semantic anomalies and d’-values for semantic anomalies in control group. (B) Correlations between 8-30 Hz ERD for syntactic anomalies and d’-values for syntactic anomalies in control group. (C) Correlations between 8-30 Hz ERD for semantic anomalies and d’-values for semantic anomalies in PPA patients. (D) Correlations between 8-30 Hz ERD for syntactic anomalies and d’-values for syntactic anomalies in PPA patients. Correlations (Spearman's Rho) between MEG task activity and factor scores for language component derived from PCA analysis for patients and controls. (E) Correlations between 8-30 Hz ERD for semantic anomalies and factor scores derived from the language component in control group. (F) Correlations between 8-30 Hz ERD for syntactic anomalies and factor scores derived from the language component in control group. (G) Correlations between 8-30 Hz ERD for semantic anomalies and factor scores derived from the language component in PPA patients. (H) Correlations between 8-30 Hz ERD for syntactic anomalies and factor scores derived from the language component in PPA patients.

For older controls, there was a significant *positive* correlation between event-related power in the 8–30 Hz frequency range and comprehension performance on the semantic anomalies in the bilateral superior (BA 6) and medial frontal regions (BA 10/9), and left paracentral lobule, extending into the postcentral gyrus and superior parietal lobule (BA 7) (Fig. 4A). This positive correlation indicates that for older age-matched controls, less ERD (i.e., *more* power in 8–30 Hz range) for semantic anomalies relative to control words in these regions was associated with better performance. For syntactic anomalies, *negative* correlations with performance were found in the left and right insula (BA 13), superior and inferior parietal lobules (BA 7/40, right middle occipital (BA 19) and fusiform gyri (BA 37), and along right precentral (BA 6) and postcentral gyri (BA 2), indicating that better accuracy was associated with greater 8–30 Hz ERD (reduced power) in these regions (Fig. 4B).

For PPA patients Fig. 4C shows correlation maps between ERD responses in the main analysis window (8–30 Hz, 0.4–1 s) and accuracy on semantic anomalies. For participants with PPA, 8–30 Hz ERD in the right posterior middle and superior temporal gyri (BA 21 and 22), inferior temporal (BA 20), fusiform (BA 37), and lingual gyri (BA 18) was predictive of higher accuracy on the semantic anomalies. Better performance accuracy was also associated with 8–30 Hz ERD in the bilateral middle and inferior occipital cortex (BA 18/19), primary visual cortex (BA 17), and cerebellum. There were no significant correlations between 8-30 Hz ERD (0.4–1 s) and accuracy on syntactic anomalies for PPA patients.

### 3.5 MEG 8–30 Hz ERD correlations with language measure derived from PCA

As described in the methods section, PCA analysis revealed two components that accounted for most of the variance in the neuropsychological scores across the cohort. Language measures loaded on the first component and tests of memory and visuo-spatial abilities loaded on the second component (see Table 2).

Fig. 4G and H shows the results of correlational analysis examining relationships between the overall language performance measure derived from PCA and 8–30 Hz ERD responses for PPA patients. Because the language component differentiated PPA patients from controls, whereas there was no significant difference between groups on the memory and visuo-spatial component, only scores for language component were included in the correlation analysis. We performed voxel-wise rank-order correlations (Spearman's Rho) across patients between the factor scores derived for the language component and ERD in the 8–30 Hz range. As with correlations with task accuracy, because ERD is a negative quantity, negative correlations reflect that greater 8–30 Hz ERD corresponds to better language performance.

For participants with PPA, there was no significant correlation between event-related power in the 8–30 Hz frequency range for semantic anomalies and factor scores on the language component (Fig. 4G). For syntactic anomalies, event-related power in the 8–30 Hz frequency range was significantly correlated with the language component in the right superior and inferior portions of parietal lobule, including precuneus (BA 7) and supramarginal gyrus (BA 40). These effects extended into the posterior cingulate gyrus (BA 31). Areas of significant correlation were also found in the right fusiform (BA 37) and middle temporal gyrus (BA 22/21). In the left hemisphere, a cluster of significant correlation was observed in the posterior superior temporal gyrus (BA 22), and extended into the left cingulate gyrus (BA 31) (Fig. 4H). There were no significant correlations for the age-matched control group.

### 3.6 Latency of 8–30 Hz responses

Visual inspection of the oscillatory power time-courses suggested that responses were delayed in PPA patients compared to the age-matched controls. To investigate these group differences in latency quantitatively, we computed peak latencies of 8–30 Hz ERD from time-courses of two virtual channels in the left hemisphere, the inferior parietal lobule (MNI coordinates: x = −42, y = −51, z = 50) and the inferior frontal gyrus (x = −46, y = 25, z = 18), from 100 to 2500 ms post-stimulus onset. This was done for each participant, separately for correct words and semantic and syntactic anomalies. The differences in peak latencies for PPA patients and age matched controls were investigated using one-way ANOVAs.

**Fig. 5:**
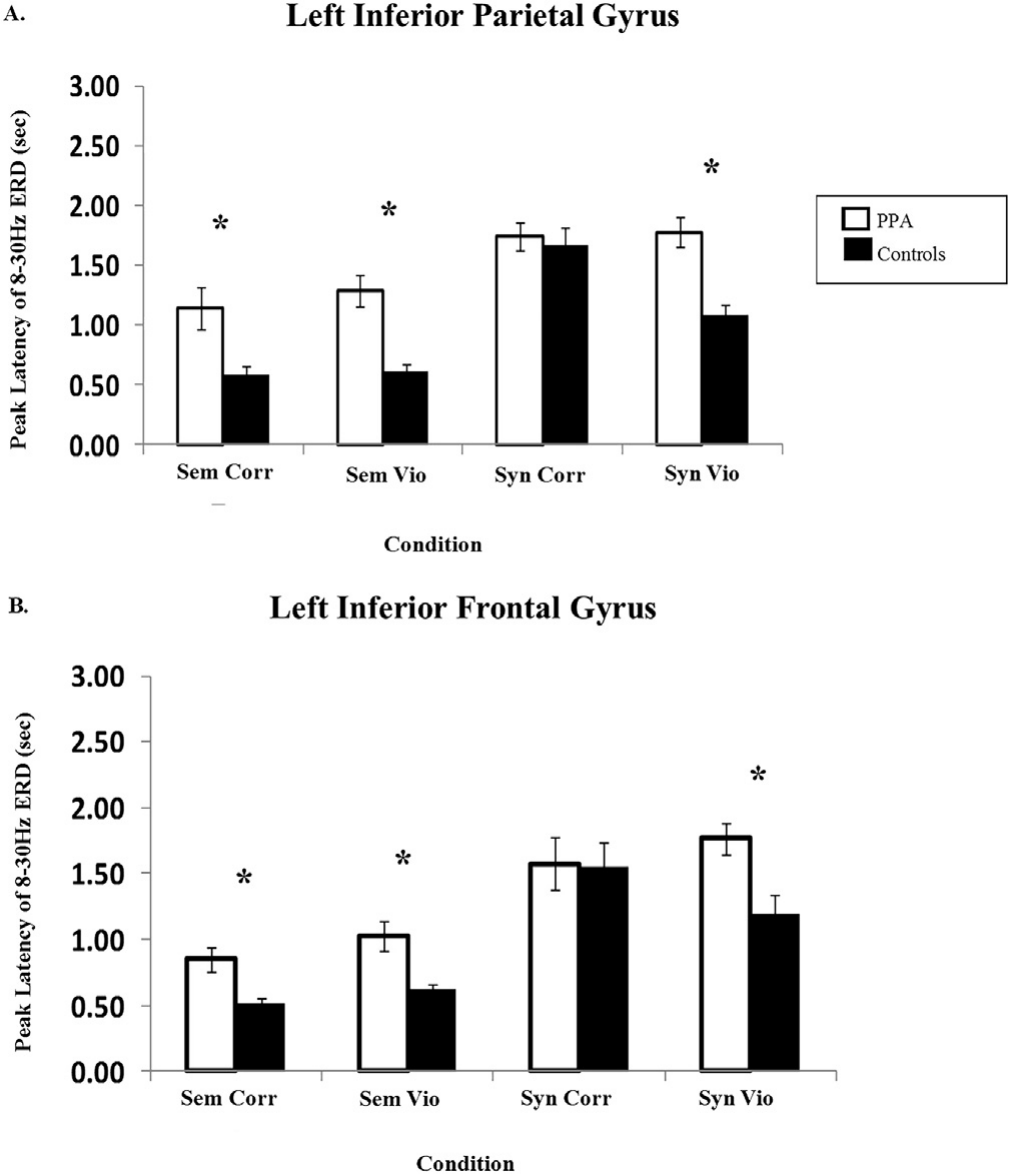
Bar charts showing peak latency of 8–30 Hz ERD responses for patients and controls in the left inferior parietal gyrus and left inferior frontal gyrus. Peak latencies were computed for each participant from 100 to 2500 ms post-stimulus onset. The four conditions shown are semantically correct words (sentence-final position), semantic violations, syntactically correct words (sentence-medial position), and syntactic violations.

Fig. 5 illustrates differences in peak latencies for patients and controls in the selected regions. The analysis revealed that PPA patients showed significantly delayed peak latencies of 8–30 Hz ERD responses compared to control participants in the left inferior parietal lobule for sentences with semantic, *F*(1, 26) = 23.301, *p* < .001, and syntactic anomalies, *F*(1, 26) = 21.15, *p* < .001. The comparison of semantic anomalies vs. correct words in the time window of 0.4–1 s for PPA patients in this region produced *8*–*30* Hz *power increase*, whereas healthy controls showed power decreases (8–30 Hz ERD). The latencies were also increased for correct semantic sentences, *F*(1, 26) = 9.49, *p* = .005, but the difference between groups was not significant for the syntactically correct, *F*(1, 26) = 0.183, *p* = .673. Visual inspection of the timecourses of oscillatory power in this region suggested that the large latency increase was responsible for the difference, as PPA patients did show ERD after an initial ERS, but with a greatly delayed latency. The responses for PPA patients were also delayed in the left inferior frontal gyrus (semantic anomalies, *F*(1, 26) = 14.26, *p* = .001; syntactic anomalies, *F*(1, 26) = 8.35, *p* = .008). The latencies were also increased for semantically correct sentences, *F*(1, 26) = 11.26, *p* = .002, but the difference between groups was not significant for the syntactically correct sentences, *F*(1, 26) = 0.004, *p* = .950.

To investigate whether delayed oscillatory responses were linked to behavioral deficits, we performed Spearman's correlations between peak latencies of 8–30 Hz ERD, d-prime values of accuracy, and reaction time. The analysis revealed that there was a significant negative relationship between peak latencies and d-prime (accuracy) for semantic anomalies in the left inferior parietal lobule, *ρ*(13) = −0.560, *p* = .046, indicating that longer latencies of 8–30 Hz ERD in this brain area were associated with lower accuracy. There was no significant relationship between latencies of 8–30 Hz ERD and d-prime values for syntactic anomalies, *ρ*(13) = −0.154, *p* = .616. In contrast to the relationship between ERD latency and accuracy (d-prime), we did not find any significant relationship between ERD latency and reaction time (RT), for either semantic, *ρ*(13) = 0.110, *p* = .720 or syntactic anomalies, *ρ*(13) = 0.283, *p* = .348. This is not surprising, however, given that the behavioral response was given after a visual cue following the end of the sentence, long after the oscillatory response to the actual anomaly is over.

## 4 Discussion

### 4.1 Altered responses to anomalies

We evaluated MEG oscillatory responses to linguistic anomalies, in patients with PPA and healthy age-matched controls. The results of the present study, consistent with our earlier studies (Kielar et al., 2016, Kielar et al., 2014) and indicate that in healthy older adults, semantic anomalies elicit left-lateralized 8–30 Hz ERD distributed more strongly along the ventral brain regions, and lasting from 400 to 1000 ms post-stimulus onset. Syntactic anomalies elicit bilateral ERD, distributed along both ventral and dorsal regions. In contrast, PPA patients showed altered patterns of language-related oscillatory responses, characterized by delayed peak latencies and attenuated amplitude. In response to semantic anomalies, the delayed latency was particularly extreme in the left inferior parietal region, resulting in an apparent 8–30 Hz power increase rather than the decrease seen in age-matched controls, for the analyzed window of 0.4–1 s. Interestingly, the distribution of this abnormal electrophysiological response did not overlap with regions of significant gray matter atrophy, which were most pronounced along the left superior and middle temporal cortex, prefrontal and inferior frontal regions.

Correlational analyses indicated that PPA patients who exhibited stronger ERD in right- hemisphere temporo-parietal areas had higher performance in detecting semantic anomalies. The 8-30 Hz ERD responses in the right temporo-parietal regions were also observed in controls. Although ERD responses in these regions were reduced in patients, the correlational results suggest that patients who retained functionality in these right hemisphere areas showed relatively preserved semantic processing. In contrast, we did not see any correlation between performance and neural activity in the left hemisphere. This pattern suggests that right-hemisphere processing of semantic information may take on an especially important role in PPA for supporting preserved comprehension abilities as the disease progresses. Interestingly, we observed a similar pattern of right-hemisphere activity related to preserved semantic comprehension in post-stroke aphasia (Kielar et al., 2016), suggesting that similar mechanisms may support residual language processing abilities in both post-stroke and progressive forms of aphasia.

The relationship between more accurate semantic performance and right hemisphere ERD in patients may be related to the more widespread distribution and bilateral organization of semantic representations, also observed in the healthy brains (Hickok and Poeppel, 2004, Hickok and Poeppel, 2007; Lambon Ralph et al., 2017). Previous studies in healthy controls suggest that the involvement of the right hemisphere homologues of the left hemisphere language regions increases as language comprehension demands become more complex (Bookheimer, 2002; Demonet et al., 2005). While the left hemisphere may be more specialized to perform fine gained coding of semantic information, coding of semantic representations is more coarse and diffuse in the right hemisphere, making it particularly well suited for tasks that place greater demands on contextual inferences, semantic integration and prediction processes (Beeman et al., 2000; Jung-Beeman, 2005; Kircher et al., 2001; Meyer et al., 2000). Recent data from semantic PPA patients suggest that the integrity of the anterior temporal lobe and left temporal pole is critical for single word comprehension. In contrast, sentence comprehension is supported by the heterogeneous network of brain regions that include left supramarginal and angular gyri, inferior frontal gyrus, dorsal premotor, and orbitofrontal cortices (Mesulam et al., 2014, Mesulam et al., 2015). Because structural integrity of the anterior temporal cortex was preserved in the current sample of nonfluent and logopenic PPA patients, semantic processing in the sentence context could be supported by the preserved functionality of the right hemisphere temporo-parietal regions.

Although patients with PPA showed significant ERD in response to syntactic anomalies, with a distribution similar to that of the control group, these regions were underactivated relative to controls, and ERD responses in these regions were not significantly correlated with better performance on the task. This indicates that in PPA, residual activity in the functionally preserved regions is not sufficient to support accurate syntactic processing. PPA patients performed very poorly on detection of syntactic anomalies, approximately at chance levels on average, whereas performance on semantic anomalies was above chance but below that seen in controls. These findings suggest that, although PPA patients retain some sensitivity to syntactic violations, this residual activity is not sufficient to process them successfully, leading to chance-level discrimination between syntactically anomalous and correct sentences. However, for age-matched controls more accurate syntactic performance was associated with recruitment of bilateral superior and inferior parietal lobules, right middle occipital and fusiform gyri, and regions along right precentral and postcentral gyri. These results suggest that under-recruitment of these regions during the sentence comprehension task may contribute to the poor syntactic performance in patients with PPA.

Although ERD responses to syntactic anomalies did not correlate with performance in detecting those anomalies (as nearly all PPA patients performed near chance levels), syntactic ERD responses were correlated with the overall level of language impairment present in the patients, as estimated by the first PCA component derived from the neuropsychological battery. The less impaired the patients were, the more strongly their brains exhibited ERD in response to syntactic violations, particularly in the right hemisphere, in both ventral and dorsal regions. This pattern suggests that even though patients were not able to detect the violations accurately, syntactic violations were still registered neurally, especially for patients with less severe language impairment. The correlation underscores the behavioral difficulty of detecting syntactic violations, and suggests that neural sensitivity as indexed by electrophysiological measurements may be a more sensitive marker of impairment than behavioral performance in sentence comprehension tasks, as anomaly detection can fall to chance levels even when the anomalies still produce a distinguishable response in the brain compared to nonanomalous words.

This pattern of results suggests that syntactic processing is uniquely vulnerable to the neuronal loss in the left perisylvian regions and may not be easily supported by the preserved brain regions in the right hemisphere alone, in contrast to semantic processing which may be adequately supported by right hemisphere engagement for much of the course of the disease.

### 4.2 Relationship with peak latencies

In comparison to the control group, PPA patients showed delayed peak latencies of 8–30 Hz ERD for semantic and syntactic anomalies. These delays in the latency of ERD responses were associated with lower task accuracy. Increased latency of electrophysiological responses during word recognition has been previously observed in PPA. In a longitudinal study of a PPA patient, Giaquinto and Ranghi (2009) found that the latencies of the N400 ERP component increased as the disease progressed. Together with the previous finding, the present results indicate that neuropathological changes in the brains of PPA patients result in slowed information processing, which is in turn linked with progressive cognitive decline. The latency of the ERD response appears to be sensitive indicator of neural dysfunction that may not be easily obtained with other neuroimaging techniques, such as fMRI or ERPs. In MRI because of slow nature of BOLD responses (on the order of seconds), the latency delay of 400 ms observed in the present study may not have a significant effect on the BOLD response. For ERPs, a prolonged latency of neural responses is likely to also increase the trial-by-trial variability of the electrophysiological response, which reduces the phase-locking of the signal and results in an attenuated amplitude and a flattened peak, making latency differences in the time-domain average signal difficult to discern. Thus, the analysis of the latency of ERD responses may be a more sensitive measure of slowed information processing resulting from cortical dysfunction in neurodegenerative disease.

### 4.3 Clinical significance

We found that electrophysiological responses to linguistic stimuli are abnormal in PPA, being attenuated in amplitude as well as delayed in latency. The participants in the study also exhibited structural atrophy in key language regions of the left hemisphere, including the inferior frontal gyrus and middle temporal lobe. However, the electrophysiological abnormalities were not limited to atrophied cortex. Reduced and delayed responses were present throughout the networks that showed sensitivity to linguistic anomalies in healthy controls. This suggests that tissue does not need to be atrophied in order for its function to be compromised in the disease. Possible mechanisms for this functional compromise include: 1) remote effects of structural damage to areas that are functionally connected to the non-atrophied regions, depriving them of their needed input or output, and 2) subtle neuronal or synaptic damage reflecting an earlier stage of disease pathology that has not yet resulted in large-scale neuronal death in the area. In fact, these two mechanisms are likely to be closely related. Recent neuropathological studies have suggested that neurodegenerative disorders characterized by abnormal protein accumulation (which describes all three recognized variants of PPA) may spread through the brain along synaptic pathways in a prion-like propagation process (Agosta et al., 2015; Duyckaerts, 2013). Thus, areas that are functionally connected to regions of atrophy are likely also to show abnormal responses in the early stages of degeneration as the proteinopathy begins to affect them. Recent studies of neural activity in PPA using resting-state recordings have also shown that functional abnormalities exist in widespread networks beyond the zones of atrophy (Bonakdarpour et al., 2017; Ranasinghe et al., 2017), and that functional connectivity in the healthy brain predicts the spread of atrophy in PPA (Mandelli et al., 2016).

Although these findings may seem like “more bad news” to patients and families struggling with the challenges of PPA, in that they indicate that dysfunction extends far beyond the regions of atrophy seen on an MRI, they also point the way to new treatment approaches that may be able to slow or reverse the progression of that dysfunction. Because new neurons are not generated in most regions of adult cortex (Lepousez et al., 2015), reversal of brain atrophy is very unlikely. Nonetheless, some interventions have been shown to ameliorate the linguistic symptoms of PPA, including language training (Evans et al., 2016; Jokel et al., 2016) and noninvasive brain stimulation (Cotelli et al., 2014; Gervits et al., 2016). Although no drugs have yet proven effective for treating PPA, the possibility remains that some could be found, especially as the mechanisms of neural dysfunction in the disease become more clear. Trials of novel interventions in PPA are difficult and expensive due to the relative rarity of the disease, the challenges that its patients have in experimental participation, and the need for long-term follow-up to test the effects of the intervention on the progression of the disease. Thus, biomarkers of the neural dysfunction that underlies the symptoms are greatly needed, to improve the sensitivity of such trials. For example, even in a patient who has become mute, an improvement in physiological parameters, such as the amplitude and latency of oscillatory responses to language stimuli, would be a sign that a given intervention has modified brain activity in the right direction. We are therefore optimistic that the increased use of neural monitoring technologies such as the MEG used in the present study will enhance the power of interventional trials to detect a therapeutic response, and will accelerate the discovery of effective symptomatic (or even disease-modifying) treatments.

### 4.4 Limitations

The limited sample size in the present study precluded the detailed characterization of differences in linguistic anomaly processing between nonfluent and logopenic PPA subtypes. We found similar patterns of oscillatory responses in both groups (Supplementary Fig. S1), and similar patterns of atrophy, despite the fact that the nonfluent and logopenic groups are known to have predominantly anterior and posterior distributions of atrophy respectively. Thus, our results can be taken as an average characterization of oscillatory responses to linguistic anomalies in PPA as a whole (excluding the semantic variant), highlighting common processes such as right temporal lobe involvement in semantic comprehension. It is likely that further studies with larger sample sizes may reveal distinct patterns of impairment and compensation in these two subtypes, with more specific implications for treatment.

### 4.5 Conclusion

Electrophysiological responses to semantic and syntactic anomalies are abnormal in PPA, being delayed in latency and attenuated in amplitude. The localization of these abnormalities extends beyond zones of atrophy, suggesting an earlier stage of functional disruption as the disease progresses. Preserved semantic processing is supported by the functionality of preserved right hemisphere regions that are also recruited in control participants. In contrast, syntactic processing seems to be more vulnerable to the neuronal loss in left hemisphere language regions, and it is less likely that activity in the preserved brain regions can successfully support syntactic computations.

The following is the supplementary data related to this article.

Fig. S1 Synthetic aperture magnetometry (SAM) maps of power changes in the 8–30 Hz frequency range and 0.4–1 s time window after critical word onset for PPA patients classified as nonfluent and logopenic variants. The maps represent average SAM pseudo-T values thresholded at 30% of the maximum response magnitude for each anomaly type. (A) Power changes for semantic anomalies vs. correct words for nonfluent PPA. (B) Power changes for semantic anomalies vs. correct words for logopenic PPA patients. (C) Power changes for syntactic anomalies vs. correct words for nonfluent PPA. (D) Power changes for syntactic anomalies vs. correct words for logopenic PPA patients. Voxel-based morphometry (VBM) maps illustrating gray matter atrophy in each PPA variant relative to controls. (E) Nonfluent PPA patients vs. controls. (F) Logopenic PPA patients vs. controls. The statistical maps are shown at a threshold of *p* < .05, uncorrected for multiple comparisons, to compare patterns of cortical atrophy in the two groups, but do not establish statistically significant differences between the two groups. Fig. S1
