# Supplementary material for: Abnormal language-related oscillatory responses in primary progressive aphasia
Source: Neuroimage Clin. 2018 Mar 1;18:560–74. doi: 10.1016/j.nicl.2018.02.028 (PMC5964832; doi:10.1016/j.nicl.2018.02.028)
Supplement: Fig. S1 — Synthetic aperture magnetometry (SAM) maps of power changes in the 8–30 Hz frequency range and 0.4–1 s time window after critical word onset for PPA patients classified as nonfluent and logopenic variants. The maps represent average SAM pseudo-T values thresholded at 30% of the maximum response magnitude for each anomaly type. (A) Power changes for semantic anomalies vs. correct words for nonfluent PPA. (B) Power changes for semantic anomalies vs. correct words for logopenic PPA patients. (C) Power changes for syntactic anomalies vs. correct words for nonfluent PPA. (D) Power changes for syntactic anomalies vs. correct words for logopenic PPA patients. Voxel-based morphometry (VBM) maps illustrating gray matter atrophy in each PPA variant relative to controls. (E) Nonfluent PPA patients vs. controls. (F) Logopenic PPA patients vs. controls. The statistical maps are shown at a threshold of p < .05, uncorrected for multiple comparisons, to compare patterns of cortical atrophy in the two groups, but do not establish statistically significant differences between the two groups. [file mmc1.docx]

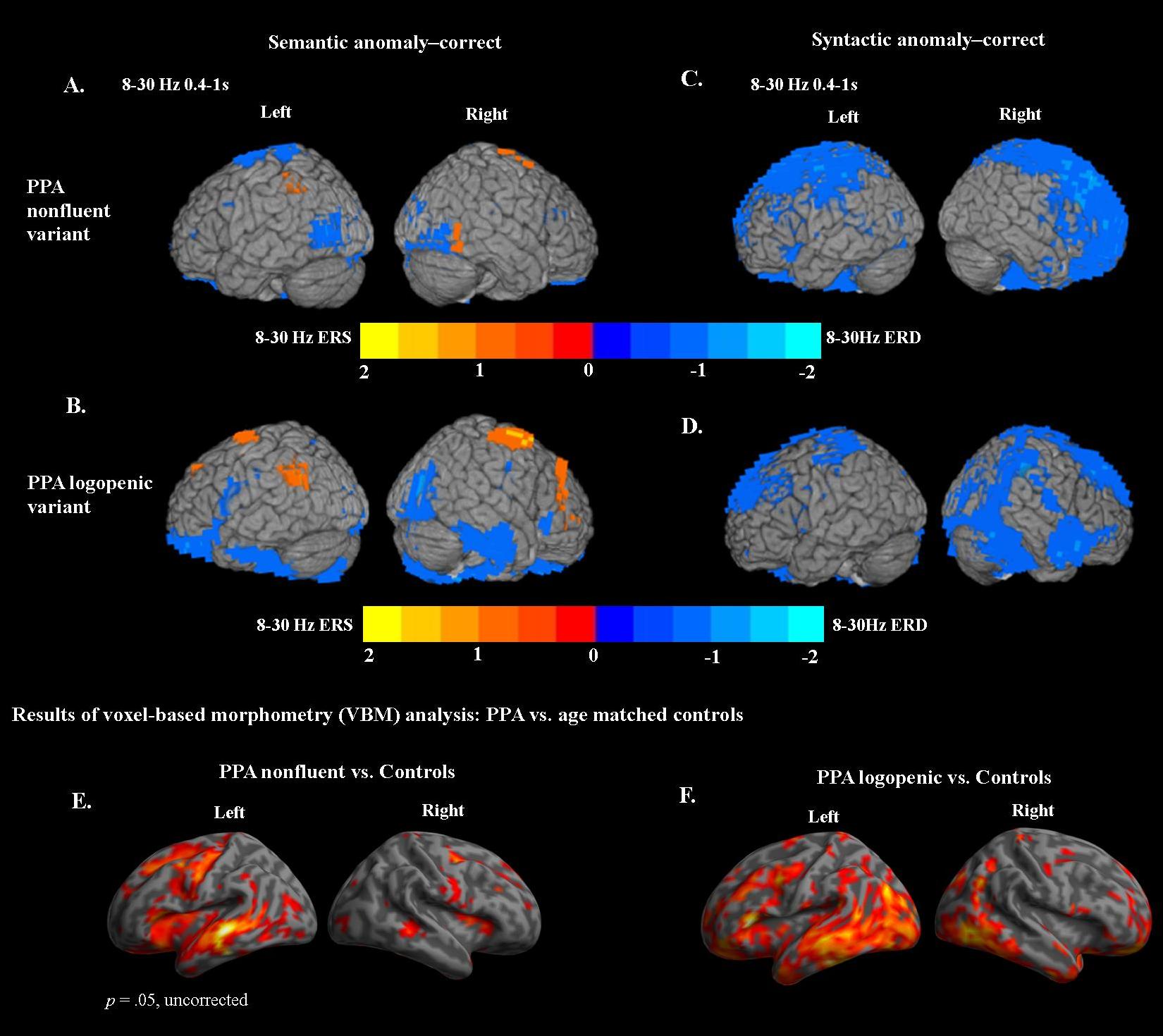


**Figure S1.** **Synthetic aperture magnetometry (SAM) maps of power changes in the 8-30 Hz frequency range and 0.4-1s time window after critical word onset for PPA patients classified as nonfluent and logopenic variants.** The maps represent average SAM pseudo-T values thresholded at 30% of the maximum response magnitude for each anomaly type. (A) Power changes for semantic anomalies vs. correct words for nonfluent PPA. (B) Power changes for semantic anomalies vs. correct words for logopenic PPA patients. (C) Power changes for syntactic anomalies vs. correct words for nonfluent PPA. (D) Power changes for syntactic anomalies vs. correct words for logopenic PPA patients. **Voxel-based morphometry (VBM) maps illustrating gray matter atrophy in each PPA variant relative to controls.** (E) Nonfluent PPA patients vs. controls. (F) Logopenic PPA patients vs. controls. The statistical maps are shown at a threshold of p<.05, uncorrected for multiple comparisons, to compare patterns of cortical atrophy in the two groups, but do not establish statistically significant differences between the two groups.
